# Mirtazapine for the Treatment of Chronic Pruritus

**DOI:** 10.3390/medicines6030073

**Published:** 2019-07-06

**Authors:** Raveena Khanna, Emily Boozalis, Micah Belzberg, John G. Zampella, Shawn G. Kwatra

**Affiliations:** 1 Department of Dermatology, Johns Hopkins University School of Medicine, Baltimore, MD 21231, USA; 2 Creighton University School of Medicine, Omaha, NE 68178, USA; 3 Department of Dermatology, New York University School of Medicine, New York, NY 10016, USA

**Keywords:** mirtazapine, chronic, pruritus, itch, refractory, treatment, noradrenergic, serotonergic, antihistaminergic, antidepressant

## Abstract

**Background:** Chronic pruritus is a debilitating condition associated with a wide range of dermatologic, systemic and psychogenic etiologies. In patients with chronic pruritus that is refractory to conventional therapy, symptoms can significantly decrease quality of life by contributing to anxiety, sleep disturbances, and in many cases depression. Recent studies have demonstrated the effectiveness of mirtazapine in relieving chronic itch that is refractory to standard first-line therapies. **Methods:** We searched PubMed for English-language articles containing the words (“pruritus” or “itch”) AND “antidepressant” and then conducted a systematic review of the current literature to summarize the efficacy of mirtazapine in treating chronic itch. **Results:** All studies reported a reduction in itch intensity following the administration of mirtazapine. **Conclusion:** Collectively, these studies suggest the potential for mirtazapine to relieve chronic itch attributed to dermatological causes and malignancies. As, such mirtazapine may be an option for patients with chronic pruritus that is refractory to typical first-line treatments.

Dear Editor,

Chronic pruritus is a common condition that can interfere with sleep and diminish overall quality of life. The current management of chronic itch is directed at the underlying cause, which can be dermatologic, systemic or psychogenic in nature [1]. First-line therapy typically begins with topical emollients, topical corticosteroids, and antihistamines. GABA-receptor modulators, opioid agonists/antagonists and phototherapy can be used for patients with refractory pruritus [1]. 

Recalcitrant itch is a distressing symptom for which a safe and effective agent is needed [2,3]. Recent studies have demonstrated the effectiveness of oral antidepressants in relieving chronic itch associated with dermatologic, systemic and psychogenic causes [4,5]. Mirtazapine, a dual noradrenergic and serotonergic antidepressant with antihistaminergic properties, is one such antidepressant that has demonstrated effectiveness in reducing itch severity. As an H_1_, 5HT_2_ and 5HT_3_-receptor blocker, mirtazapine may be an alternative therapy for pruritus that is refractory to first-line therapies. Mirtazapine is believed to centrally reduce itch by antagonizing a2-adrenergic receptors [6,7]. Aside from having a wide therapeutic index, mirtazapine is rarely known to cause the initial anxiety and nausea associated with other antidepressants effective in treating chronic itch [2,8]. To assess the efficacy of mirtazapine for the treatment of chronic pruritus, we therefore performed a systematic review of the current literature using PubMed for English-language articles containing the words (“pruritus” or “itch”) AND “antidepressant.” The Preferred Reporting Items for Systematic Reviews and Meta-Analysis (PRISMA) flow chart is shown in Figure 1. All studies reported a reduction in itch intensity following the administration of mirtazapine. Collectively, these studies suggest the potential for mirtazapine to relieve chronic itch attributed to dermatological causes and malignancies (Table 1).

There are several limitations to this review. Of the studies evaluated, most were case series or case reports. Larger, randomized controlled trials are still needed to draw definitive conclusions regarding mirtazapine’s effectiveness in reducing itch. The studies included used a wide variety of outcome measures to evaluate itch intensity, which limits our ability to directly compare outcomes. Given the significant psychological burden of chronic pruritus, the placebo effect may have also affected perceived outcomes [9]. 

Mirtazapine is currently FDA-approved for the treatment of major depressive disorder. The main side effects of mirtazapine are heavy sedation, weight gain and hypercholesterolemia [10]. Mirtazapine is contraindicated in patients taking monoamine oxidase inhibitors (MAOIs) given the increased risk of serotonin syndrome [6]. Prior to prescribing mirtazapine, physicians should obtain a baseline lipid panel, liver function tests, and fasting blood glucose levels [11]. The FDA-approved starting dose for mirtazapine is 15 mg orally every night for the treatment of major depressive disorder (MDD) [7,11]. Physicians should counsel patients to report any signs of worsening depression or suicidal ideations upon the initiation of treatment or as a result of dosage changes. It is also advised that physicians schedule a follow-up appointment six-weeks after initiating treatment to evaluate for clinical improvement, drug reactions, or adverse effects. 

In conclusion, mirtazapine may be an option for patients with chronic pruritus that is refractory to typical first-line treatments, but future randomized controlled trials are needed to determine the efficacy of therapy, optimal dosing regimens, and the types of chronic pruritus that benefit most from treatment with oral antidepressants.

## Figures and Tables

**Figure 1:**
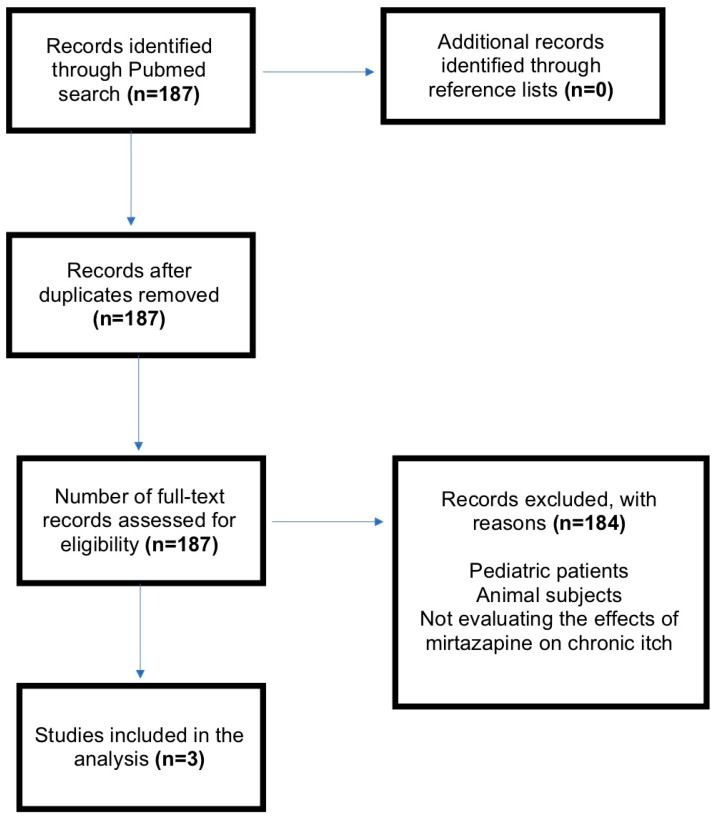
Preferred Reporting Items for Systematic Reviews and Meta-Analyses (PRISMA) flow-chart. *n*, number of articles; *Mirtazapine*.

**Table 1:** Summary of studies examining the effects of mirtazpine in treating chronic pruritus. * Determined based on the Oxford Centre for Evidence-based Medicine (CEBM) Levels of Evidence. *Mirtazapine*; *CS*, case series; *CR*, case report.

Grade of Recommendation *	Study Design	Diagnosis	N	Regimen	Degree of Pruritus Reduction	Reference
C	CS	Atopic dermatitis	2	15 mg mirtazapine every day for three months (first patient) and six months (second patient).	Both patients noted a “significant reduction” of nocturnal and daytime pruritus and improvement in sleep.	[7]
		Lichen simplex chronicus	1	15 mg mirtazapine every day for two months.	Patient noted a complete resolution of nocturnal pruritus and “significant reduction” in daytime pruritus.	
C	CS	Adenocarcinoma of unknown origin	4	15 mg mirtazapine every night until death.	The patient reported complete resolution of pruritus within 24 hours.	[2]
		Nodular sclerosis Hodgkin’s disease		15 mg mirtazapine every night. After an unspecified length of time, the dosage was increased to 30 mg every night until death.	The patient reported 75% improvement in pruritic symptoms on 15 mg mirtazapine and complete resolution of itch on 30 mg mirtazapine.	
		Large B-cell lymphoma		15 mg mirtazapine every night. After an unspecified length of time, the dosage was increased to 30 mg every night and continued for an unspecified length of time.	The patient reported 80% improvement in pruritic symptoms on 15 mg mirtazapine and complete resolution of itch on 30 mg mirtazapine.	
		Advanced renal cell carcinoma		15 mg mirtazapine every night. One month later, dosage was increased to 30 mg and maintained at that level until death.	The patient reported complete resolution of pruritus on 30 mg mirtazapine.	
C	CR	Carcinoma en cuirasse	1	15 mg oral mirtazapine every night for an unspecified duration.	The patient reported a decrease in itch intensity from “200/100” pre-treatment to 2/100 twelve hours after starting mirtazapine.	[6]
